# α-Diimine Cisplatin Derivatives: Synthesis, Structure, Cyclic Voltammetry and Cytotoxicity

**DOI:** 10.3390/molecules27238565

**Published:** 2022-12-05

**Authors:** Dmitriy S. Yambulatov, Irina A. Lutsenko, Stanislav A. Nikolaevskii, Pavel A. Petrov, Ivan V. Smolyaninov, Irina K. Malyants, Victoria O. Shender, Mikhail A. Kiskin, Alexey A. Sidorov, Nadezhda T. Berberova, Igor L. Eremenko

**Affiliations:** 1 N. S. Kurnakov Institute of General and Inorganic Chemistry, Russian Academy of Sciences, 31 Leninsky Prosp., 119991 Moscow, Russia; 2 Nikolaev Institute of Inorganic Chemistry, Siberian Branch of the Russian Academy of Sciences, 630090 Novosibirsk, Russia; 3 Department of Chemistry, Astrakhan State Technical University, 16 Tatisheva Str., 414056 Astrakhan, Russia; 4 Federal Research and Clinical Center of Physical-Chemical Medicine of Federal Medical Biological Agency, 1a M. Pirogovscaya, 119435 Moscow, Russia

**Keywords:** platinum(II), redox-active ligand, 1,4-diaza-1,3-butadienes, cyclic voltammetry, α-diimine, bioinorganic chemistry, cisplatin

## Abstract

Three new Pt(II) complexes [(dpp-DAD)PtCl_2_] (**I**), [(Mes-DAD(Me)_2_)PtCl_2_] (**II**) and [(dpp-DAD(Me)_2_)PtCl_2_] (**III**) were synthesized by the direct reaction of [(CH_3_CN)_2_PtCl_2_] and corresponding redox-active 1,4-diaza-1,3-butadienes (DAD). The compounds were isolated in a single crystal form and their molecular structures were determined by X-ray diffraction. The purity of the complexes and their stability in solution was confirmed by NMR analysis. The Pt(II) ions in all compounds are in a square planar environment. The electrochemical reduction of complexes **I**–**III** proceeds in two successive cathodic stages. The first quasi-reversible reduction leads to the relatively stable monoanionic complexes; the second cathodic stage is irreversible. The coordination of 1,4-diaza-1,3-butadienes ligands with PtCl_2_ increases the reduction potential and the electron acceptor ability of the DAD ligands. The synthesized compounds were tested in relation to an adenocarcinoma of the ovary (SKOV3).

## 1. Introduction

Bioinorganic chemistry is one of the most promising areas in coordination chemistry. Based on the amount of accumulated data, it can be confidently stated that the studied metal containing compounds can become promising metallodrugs [1,2,3,4] similarly to cis-diamminedichloroplatinum (cisplatin, CP) [5]—the first FDA-approved platinum compound for treating cancer, which is currently prescribed to almost half of cancer the patients worldwide. However, serious side effects [6,7,8,9], as well as congenital or acquired resistance to CP [10,11], provoked an active search for alternative platinum preparations, but with a different combination of ligands.

Among the promising organic components are *red-ox* active or *non-innocent* ligands capable of reversibly accepting electrons [12]. Over the past two decades, it has become clear that redox-active systems based on non-innocent ligands and metals with switchable oxidation states play a crucial role in biological processes [13,14,15], the generation of free radicals can be used for treating of many diseases [16]. One of the most studied redox-active ligands are α-diimines or 1,4-diaza-1,3-butadienes [17,18,19,20]. Compounds of such ligands are mainly investigated for creating efficient catalysts [21,22,23,24,25], small molecules activation [26,27,28] and switchable materials [29,30,31,32,33,34,35]. It has recently been found that platinum and palladium α-diimine complexes reveal cytotoxicity against various cancer cells [36,37].

In searching for new homologues of cisplatin, we prepared three platinum(II) chloride complexes with substituted 1,4-diaza-1,3-butadienes, determined their structures and investigated their electrochemical properties and cytotoxicity compared with cisplatin.

## 2. Results

### 2.1. Synthesis and Characterization of [(dpp-DAD)PtCl_2_] (**I**), [(Mes-DAD(Me)_2_)PtCl_2_] (**II**) and [(dpp-DAD(Me)_2_)PtCl_2_] (**III**)

**Scheme 1:**
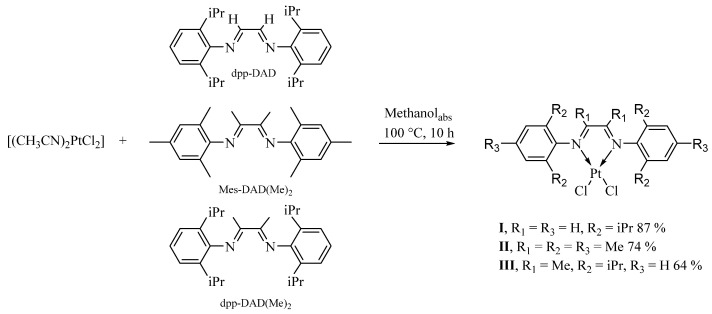
Synthesis of complexes **I**–**III**.

The reaction between presynthesized acetonitrile Pt(II) chloride complex with substituted redox-active 1,4-diaza-1,3-butadienes in absolute methanol led to the formation of three new Pt(II) diimine compounds (Scheme 1). The reactions proceed in an inert atmosphere in a sealed thick-wall glass ampoules on heating with color changing from initial yellow to brown-red. The products crystallize with further cooling of the reaction mixture to room temperature without concentration. Decanted and twice washed, the major products were isolated in 64–87% yields. We found that the reaction is best carried out in an inert atmosphere in absolute methanol, whereas the same reactions in air in non-absolute solvents decrease product yields.

In the IR spectra (see Supplementary Information) of **I**–**III**, the absorption bands at 1588 cm^−1^ (**I**), 1601 cm^−1^ (**II**), 1588 cm^−1^ (**III**) correspond to C=N stretching vibrations of the neutral diimine ligands with a slight decrease in frequencies compared to free 1,4-diaza-1,3-butadienes [17,38]. The band-shift to the lower wavenumbers indicates the coordination of two α-diimine nitrogen atoms [39].

^1^H and ^13^C NMR spectra (see Supplementary Information) are in agreement with the structural data. The signal of vinyl protons in ^1^H NMR spectrum of **I** is shifted downfield compared to free ligand (8.81 and 8.11 ppm, respectively). Additionally, due to coupling on ^195^Pt nuclei, satellites are observed with ^3^*J* (^1^H,^195^Pt) of 91.4 Hz. In ^13^C NMR spectra of **I** and **III**, signals of imine carbon atoms are also shifted downfield compared to free ligands by 3 and 8 ppm, respectively. ^13^C NMR spectrum of **II** could not be obtained due to the low solubility of the compound in the common deuterated solvents.

### 2.2. Molecular Structures of **I** and **II**

**Figure 1:**
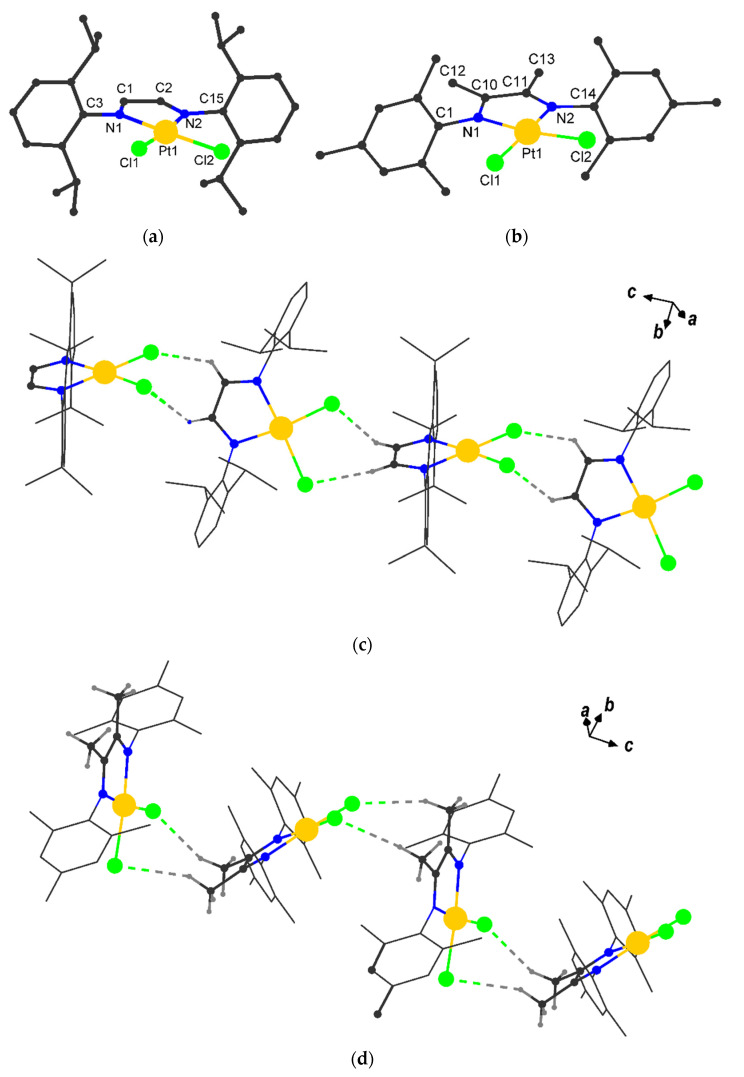
Molecular structures and crystal packing (along *c* axis) of **I** (**a**,**c**) and **II** (**b**,**d**). Hydrogen atoms are omitted for clarity, H…Cl interactions are dashed.

Complexes **I** and **II** crystallize in the monoclinic space groups *P2_1/c_* and *Cc*, respectively; experimental and crystal data are presented in Table 1. Compound **I** is isostructural to known palladium complex [40]. Both Pt(II) compounds are mononuclear, where the central platinum(II) ion coordinates two chloride anions and two nitrogen atoms of α-diimine moiety (Figure 1a,b) forming a square planar environment (selected bond distances and angles for **I** and **II** given in Table 2), which is quite typical for Pt^2+^, e.g., there is the same environment in starting [(CH_3_CN)_2_PtCl_2_] [41] and similar platinum(II) complexes [L_2_PtCl_2_], where L is N,N-dimesityl-1,4-diazabutadiene, *N*,*N*’-bis(4-hydroxyphenyl)ethylenediamine [37,42].

Analysis of the crystal packing revealed intermolecular non-covalent interactions H…Cl (the interatomic distance is shorter than the sum of Bondi’s vdW radii (2.95 Å) [43]) for both compounds (Table S1), which are responsible for the formation of supramolecular chains. In **I** the chain has a zigzag shape, which is the result of an asymmetric H…Cl contacts of molecules, the angle between the PtN_2_Cl_2_ planes is 69.88(7)° (Figure 1c). In the crystal **II** adjacent molecules form zigzag chain (Figure 1d), the angle between the PtN_2_Cl_2_ planes is 62.4(2)°.

### 2.3. Cyclic Voltammetry of **I**–**III**

**Scheme 2:**
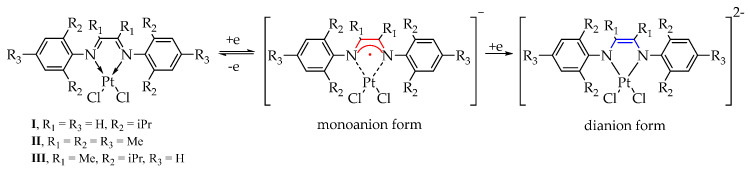
Two successive electrochemical oxidation stages for complexes **I**–**III**.

Complex 1,4-Diaza-1,3-butadienes are non-innocent ligands which can form three different redox states: neutral diimine (L^0^), radical anion (L^−^) and dianion (L^2−^) (Scheme 2). The electrochemical studies allow to observe the existence of the different redox transitions under the conditions of electrochemical experiment. The presence of redox-active ligands in the compounds **I**–**III** determines the possibility of their participation in the electrochemical transformations.

The electrochemical behavior of platinum complexes **I**–**III** was studied by cyclic voltammetry in dichloromethane solutions using a GC working electrode (Table 3).

**Figure 2:**
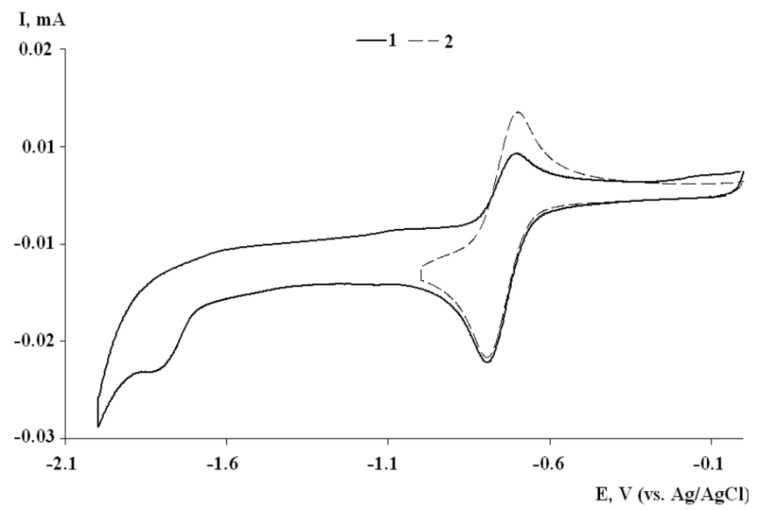
Cyclic voltammograms of **II**: the potential switch from 0.0 to −2.0 V (curve 1); the potential switch from 0.0 to −1.0 V (curve 2) (CH_2_Cl_2_, C = 2 mmol, 0.1 M TBAP, scan rate 200 mV∙s^−1^).

**Figure 3:**
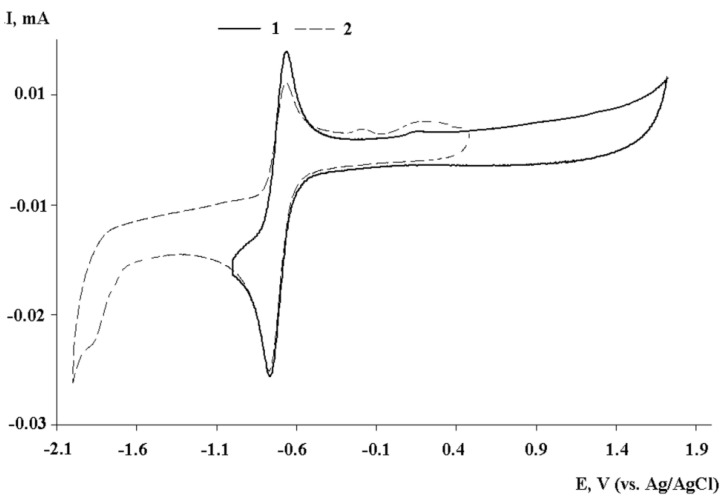
Cyclic voltammograms of **III**: the potential switch from 1.7 to −1.0 V (curve 1); the potential switch from 0.5 to −2.0 V (curve 2) (CH_2_Cl_2_, C = 2 mmol, 0.1 M TBAP, scan rate 200 mV∙s^−1^).

The electrochemical reduction of complexes **I**–**III** proceeds in two successive cathodic stages (Figure 2, Figure 3 and Figure S9). For all complexes, the first cathodic peak is quasi-reversible and one-electron, indicating the formation of monoanionic species relatively stable over the CV experiment time.

Based on the values of current ratios (I_a_/I_c_), monoanionic complexes formed during the electroreduction of compounds **I** and **III**, containing *iso*-propyl groups in the positions of 2,6-aromatic ring, have the highest stability than complex **II** with mesityl substituents at nitrogen atoms. The second reduction stage for complexes **I**–**III** is irreversible, which suggests the formation of an unstable intermediate (Scheme 2).

The high stability of the electrogenerated monoanionic complexes and the absence of the chloride reoxidation peak (1.40–1.50 V) on the reverse scans of the CVs (Figure 3), which can be formed upon metal reduction, confirmed the ligand participation in the redox processes. An increase in the potential sweep range (up to −2.0 V) with the capture of the second cathodic peak led to a decrease of the anodic current intensity of the first reduction peak (Figure 2, Figure 3 and Figure S9). This fact reveals the occurrence of a chemical stage following the second electron transfer. The electrogenerated dianion forms of complexes are unstable and possessed the basic properties. Additional peaks are observed on the reverse branch of CV in the potential range from −0.1 to 0.1 V (Figure 3).

It is worth noting that the complex **I** with dpp-DAD ligand undergoes one-electron reduction at the potential (−0.40 V) shifting to the anode potential range compared with other compounds. This behavior is due to the absence of donor methyl groups in the N = CH-CH = N moiety. A similar effect is observed for the reduction peaks of free ligands: −2.07 V (dpp-DAD(Me)_2_) [44], −1.74 V (*Mes*-DAD(Me)_2_) (Figure S10); *dpp*-DAD [44]. As in the case of the previously discussed complexes of *o*-(imino)benzoquinones with metal halides [45], the coordination of diazadiene ligands with PtCl_2_ increased in the reduction potential and, consequently, the electron acceptor ability of the 1,4-diaza-1,3-butadiene ligands. The complex formation favors not only to a shift in the reduction potential of the bound ligand but also hinders the oxidation stage. In the anode potential range, no additional peaks were observed up to 2.0 V (Figure S11).

The first reduction potential of the DAD ligands, being coordinated on the platinum(II), shifts significantly toward the anodic region compared to the corresponding free ligand. This value is 1.3 V for complexes **I** and **III**, while for compound **II** it is equal 0.97 V. The coordination of dpp-DAD with ZnCl_2_ led to a shift in the reduction potential of the ligand up to −0.54 V [44]. In case of (Mes-DAD(Me)_2_)AlCl_2_ complex containing the monoanionic form of the ligand, the potential of redox transition (L^−^/L^0^) also becomes more positive (−0.2 V, vs. Ag/AgCl) [46]. The value of the reduction potential shift depends on the Lewis acidity of the metal halides.

Thus, according to CV data, the coordination of DAD ligands to PtCl_2_ leads to a significant reduction potential shift to the anode range. This trend indicates an increase in oxidizing properties compared with the neutral 1,4-diaza-1,3-butadiene ligands.

### 2.4. Biological Activity of **I**–**III**

**Figure 4:**
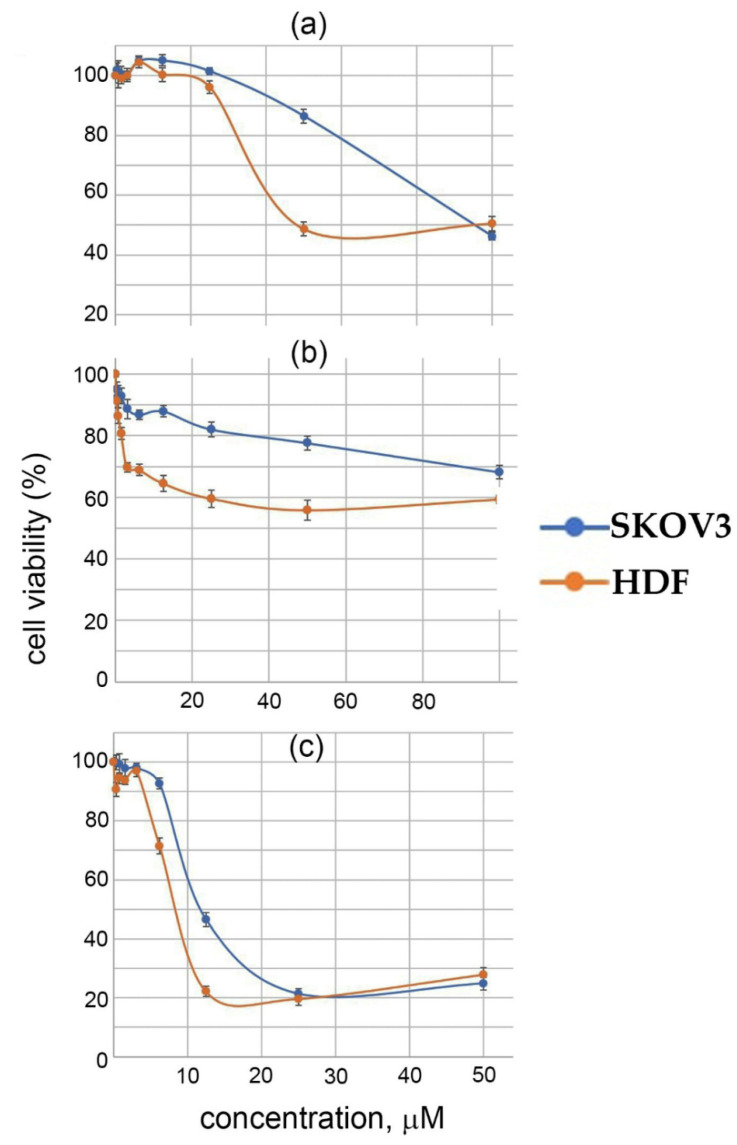
Survival rates of the SKOV3 (blue) and HDF (orange) cells incubated with various concentrations of complexes **I** (**a**)**, II** (**b**) and **III** (**c**) dissolved in DMSO. The mean value of the MTT index ± standard deviation calculated from three independent measurements is given.

Biological tests were performed on ovarian adenocarcinoma SKOV3 for **I**–**III**. The cytotoxicity of the complexes was determined against SKOV3 human ovarian adenocarcinoma cells and a primary culture of HDF human fibroblasts as a non-tumor control. Based on the MTT test data, the half-maximal inhibition dose (IC50) for each substance was calculated. Promising drugs are those that cause the death of tumor cells at minimal concentrations, while, to a lesser extent, disrupting the viability of normal cells. The IC50 for SKOV3 and HDF are presented in Table 4 and in Figure 4.

The complex **III** is similar in activity to CP, but more toxic to healthy HDF (Figure 4c), which limits the possibility of its use for therapeutic purposes. Compounds **I** and **II** (Figure 4a,b) demonstrated, at low biological activity, practical safety for healthy fibroblasts.

## 3. Materials and Methods

### 3.1. General Remarks

Synthetic procedures of obtaining new compounds were made in an inert atmosphere using sealed vacuumed glass ampoules or standard Schlenk technique. The synthesized products are stable to the oxygen and moisture. Initial Pt(II) compound [(CH_3_CN)_2_PtCl_2_] was prepared from K_2_PtCl_4_ in a water-acetonitrile solution (in a 1:1 ratio). Acetonitrile was used as received. 1,4-diaza-1,3-butadienes Pt(II) complexes were synthesized in a methanol, the latter was dried with sodium metal and stored on the molecular sieves (4 Å), condensed to the reaction mixture prior to use. The 1,4-Bis(2,6-diisopropylphenyl)-1,4-diazabuta-1,3-diene (dpp-DAD) [47], 1,4-Bis(2,4,6-trimethylphenyl)-2,3-dimethyl-1,4-diazabuta-1,3-diene (Mes-DAD(Me)_2_) [48], and 1,4-Bis(2,6-diisopropylphenyl)-2,3-dimethyl-1,4-diazabuta-1,3-diene (dpp-DAD(Me)_2_) [23] were synthesized with similar procedures as reported earlier. Product yields were calculated relative to the initial number of ligands.

IR spectra of the compounds were recorded in the range 400–4000 cm^−1^ on a Perkin Elmer (Waltham, MA, USA) Spectrum 65 spectrophotometer equipped with Quest ATR Accessory (Specac (Orpington, Kent, UK)) using the method of attenuated total reflection (ATR). NMR spectra were recorded on a Bruker Avance III 500 FT spectrometer with operating frequencies of 500.03 and 125.73 MHz for ^1^H and ^13^C nuclei, respectively. Chemical shifts are reported in ppm of the δ scale and relate to signals of the solvent.

X-ray diffraction studies of single crystals of complexes **I** and **II** were performed on a Bruker D8 Venture (Bruker, Billerica, MA, USA) with a CCD camera and a graphite monochromated MoKα radiation source (λ = 0.71073 Å). Semiempirical absorption corrections were applied for all the experiments using SADABS (University of Gottingen, Gottingen, Germany) [49]. The structures were solved by direct methods and refined in the full-matrix anisotropic approximation for all non-hydrogen atoms. Hydrogen atoms at carbon atoms of organic ligands were generated geometrically and refined in the “riding” model. The calculations were performed in SHELX [50] using Olex2 (OlexSys Ltd., Chemistry Department, Durham University, DH1 3LE, UK) [51]. The main crystallographic parameters and refinement details of compounds **I** and **II** are listed in Table 2. The structure parameters were deposited with the Cambridge Structural Database (CCDC Nos. 2216696 (**I**) and 2216697 (**II**); deposit@ccdc.cam.ac.uk or http://www.ccdc.cam.ac.uk/data_request/cif, accessed on 13 October 2022).

Cyclic voltammetry studies were carried out using VERSASTAT-3 potentiostat (PAR) in three-electrode mode. The stationary glassy carbon (d = 2 mm) disk was used as the working electrode; the auxiliary electrode was the platinum-flag electrode. The reference electrode was Ag/AgCl/KCl (sat.) with a watertight diaphragm. All measurements were carried out under argon. The samples were dissolved in the pre-deaerated solvent. The scan rate (ν) was 200 mV∙s^−1^. The supporting electrolyte 0.1 M Bu_4_NClO_4_ was dried under reduced pressure (48 h) at 50 °C. The concentration of compounds was 1–3 mmol.

The cytotoxic effect of various concentrations of the **I**, **II** and **III** complexes on the SKOV3 human ovarian adenocarcinoma cell line and human dermal fibroblast (HDF) primary culture was measured using the MTT assay. This test is based on measuring the activity of the mitochondrial enzyme succinate dehydrogenase and is widely used to evaluate the anticancer activity of potential drugs in vitro. From the MTT test, the half-maximal inhibition dose (IC50) was calculated for both substances. SKOV3 cells were obtained from the ATCC collection, the primary HDF culture was obtained from a healthy donor. SKOV3 and HDF cells were cultured in DMEM (10% FBS, 2 mM glutamine, 1% gentamicin). The cells were cultivated in plastic flasks under sterile conditions, the cells were incubated at 37 °C in 5% CO_2_.

The stock solutions (50 mM) of compounds **I**, **II** and **III** were prepared in DMSO; before adding to the cells, they were diluted to the required concentrations in a culture medium, and SKOV3 and HDF were seeded in the wells of 96-well plates in the amount of 4 × 10^3^ cells per well and 3.5 × 10^3^ cells per well, respectively. The cells were allowed to fix for 14 h, after which different concentrations of the test compounds or DMSO (as a control sample) were injected in triplicate by the titration method. The final volume of the medium in the wells was 100 μL. At the end of the period of 48 h after the addition of samples, the cell viability was measured using the MTT reagent (Sigma). The 10 μL volume of an MTT working solution (7 mg/mL) was added to each well with cells (100 μL of a medium) and the wells were incubated for 3 h, after which the medium was replaced with a DMSO solution. Using a tablet spectrophotometer (TECAN Infinite M Plex), the absorbance of each well was determined at 570 nm with subsequent subtraction of the background absorbance. The concentration value and the IC50 inhibition dose were determined from the dose–response curves.

### 3.2. Synthesis of [(dpp-DAD)PtCl_2_] (**I**)

Weighed portions of [(CH_3_CN)_2_PtCl_2_] (0.347 g, 1.000 mmol) and dpp-DAD (0.376 g, 1.0 mmol) were placed in a thick-walled glass ampoule (V = 100 mL) and degassed in a dynamic vacuum with a cold trap for 30 min, absolute methanol (70 mL) was condensed into the ampoule, the latter was fire-sealed and heated in an oil bath (100 °C, *caution—high pressure!*) for 10 h until the all-yellow platinum(II) chloride acetonitrile complex dissolved. The reaction mixture color changed from initial yellow to brown-red. Red prism crystals of the product were grown on the ampoule walls during heating. The product was isolated by further cooling to RT and decantation, twice washed with cold methanol (0.558 g, 87%). Anal. calcd for C_26_H_36_Cl_2_N_2_Pt (641.19) C, 48.60; H, 5.65; N, 4.36. Found: C, 48.44; H, 5.59; N, 4.32. IR, ν/cm^−1^: 3071 w, 3027 w, 2958 vs, 2927 s, 2871 m, 1588 w, 1519 m, 1436 s, 1384 w, 1356 w, 1328 m, 1259 m, 1259 m, 1184 m, 1105 m, 1055 m, 939 w, 873 m, 797 vs, 781 s, 701 m, 644 w, 594 m, 544 w, 462 s.

^1^H NMR (CDCl_3_): δ 8.81 (s, 2H, HC=N), 7.44 (m, 2H, *p*-CH), 7.32 (m, 4H, *m*-CH), 3.24 (sept, ^3^*J*_H–H_ = 6.8 Hz, 4H, CH(CH_3_)_2_), 1.44 (d, ^3^*J*_H–H_ = 6.8 Hz, 12H, CH(CH_3_)_2_), 1.21 (d, ^3^*J*_H–H_ = 6.8 Hz, 12H, CH(CH_3_)_2_). ^13^C NMR (CDCl_3_): δ 166.3, 144.0, 141.1, 129.8, 124.6, 28.7, 24.6, 23.2.

### 3.3. Synthesis of [(Mes-DAD(Me)_2_)PtCl_2_] (**II**)

The complex was obtained by a similar method of **I**. Weighed portions of [(CH_3_CN)_2_PtCl_2_] (0.347 g, 1.000 mmol) and Mes-DAD(Me)_2_ (0.320 g, 1.0 mmol) were placed in a thick-walled glass ampoule (V = 100 mL) and degassed in a dynamic vacuum with a cold trap for 30 min, absolute methanol (70 mL) was condensed into the ampoule, the latter was fire-sealed and heated in an oil bath (100 °C, *caution—high pressure!*) for 10 h until the all-yellow platinum(II) chloride acetonitrile complex dissolved. The solution color changed from initial yellow to brown. The reaction mixture was slowly cooled (10 °C/h) to RT resulted the formation of brown needle-like crystals (0.432 g, 74%). Anal. calcd for C_22_H_28_Cl_2_N_2_Pt (585.13) C, 45.06; H, 4.81; N, 4.78. Found: C, 45.02; H, 4.76; N, 4.63. IR, ν/cm^−1^: 3266 w, 3212 w, 3134 w, 3012 w, 2968 w, 2911 w, 2849 w, 1601 m, 1579 m, 1475 s, 1456 s, 1434 s, 1381 s, 1337 m, 1237 m, 1209 w, 1146 m, 990 s, 848 vs, 804 w, 757 w, 716 w, 572 m, 512 w, 465 w. ^1^H NMR (CDCl_3_): δ 7.00 (m, 4H, *m*-CH), 2.34 (s, 6H, *p*-CH_3_), 2.24 (s, 12H, *o*-CH_3_), 1.26 (s, 6H, N=C–CH_3_).

### 3.4. Synthesis of [(dpp-DAD(Me)_2_)PtCl_2_] (**III**)

The complex was obtained by a similar method of **I** and **II**. Weighed portions of [(CH_3_CN)_2_PtCl_2_] (0.347 g, 1.000 mmol) and dpp-DAD(Me)_2_ (0.404 g, 1.0 mmol) were placed in a thick-walled glass ampoule (V = 100 mL) and degassed in a dynamic vacuum with a cold trap for 30 min, absolute methanol (70 mL) was condensed into the ampoule, the latter was fire-sealed and heated in an oil bath (100 °C, *caution—high pressure!*) for 10 h until the all-yellow platinum(II) chloride acetonitrile complex dissolved. The reaction mixture color changed from initial yellow to red-brown. The solution was slowly cooled (10 °C/h) to RT resulted the formation of brown needle-like crystals (0.428 g, 64%). Anal. calcd for C_28_H_40_Cl_2_N_2_Pt (669.22) C, 50.15; H, 6.01; N, 4.18. Found: C, 50.07; H, 5.59; N, 4.13. IR, ν/cm^−1^: 3074 w, 3024 w, 2959 vs, 2924 s, 2867 m, 1589 w, 1522 w, 1450 s, 1383 vs, 1326 s, 1255 m, 1221 m, 1179 m, 1099 w, 1057 m, 996 s, 931 m, 792 vs, 740 s, 704 w, 641 w, 570 w, 538 w, 477 w, 449 m, 404 m. ^1^H NMR (CDCl_3_): δ 7.39 (m, 2H, *p*-CH), 7.30 (m, 4H, *m*-CH), 3.07 (sept, ^3^*J*_H–H_ = 6.8 Hz, 4H, CH(CH_3_)_2_), 1.66 (s, 6H, N=C–CH_3_), 1.45 (d, ^3^*J*_H–H_ = 6.8 Hz, 12H, CH(CH_3_)_2_), 1.20 (d, ^3^*J*_H–H_ = 6.8 Hz, 12H, CH(CH_3_)_2_). ^13^C NMR (CDCl_3_): δ 168.4, 141.2, 140.2, 129.4, 124.0, 28.8, 23.9, 23.8, 20.7.

## 4. Conclusions

Thus, here we present the synthesis and characterization of three new platinum(II) compounds with α-diimine ligands. All of them were characterized with ^1^H and ^13^C NMR, IR-spectra and elemental analysis. For [(dpp-DAD)PtCl_2_] (**I**) and [(Mes-DAD(Me)_2_)PtCl_2_] (**II**) crystal structures were determined reveal square planar geometry of N_2_PtCl_2_ moiety. The complexes **I**–**III** were characterized by electrochemistry. The cyclic voltammetry of **I**–**III** showed two reduction processes. The first cathodic stage was quasi-reversible and one-electron, indicating the formation of monoanionic species. The second reduction step was irreversible. The coordination of DAD ligands to PtCl_2_ led to the significant reduction potentials shift to anode range compared with the free DAD ligands. Compounds **I** and **II** demonstrated low biological activity against SKOV3 human ovarian adenocarcinoma cells, whereas [(dpp-DAD(Me)_2_)PtCl_2_] (**III**) is similar in activity to cisplatin and toxic to healthy fibroblasts. Complexes **I** and **II** are safe for healthy cells.

## Figures and Tables

**Table 1:** Crystallographic data, details of data collection, and characteristics of data refinement for **I** and **II**.

Parameter	Value	
	I	II
Molecular formula	C_26_H_36_Cl_2_N_2_Pt	C_22_H_28_Cl_2_N_2_Pt
*Mw*, g mol^−1^	642.56	586.45
*T*, K	293	100
Crystal system	Monoclinic	Monoclinic
Space group	*P*2_1_/*c*	*Cc*
*a*, Å	13.9314(13)	22.738(3)
*b*, Å	15.2288(14)	7.2591(8)
*c*, Å	14.0105(15)	13.5245(15)
*V*, Å^3^	2759.6(5)	2216.1(4)
*β*, deg	111.816(4)	96.903(4)
*Z*	4	4
ρ_calcd_, g cm^−3^	1.547	1.758
μ, mm^−1^	5.293	6.582
θ range, deg	2.06–30.72	2.79–30.59
*F*(000)	1272	1144
Index range	–20 ≤ *h* ≤ 19; –21 ≤ *k* ≤ 21; –19 ≤ *l* ≤ 19	–31 ≤ *h* ≤ 31; –10 ≤ *k* ≤ 9; –18 ≤ *l* ≤ 18
Number of reflections collected	28,952	20,002
Number of unique reflections	8375	5791
*R_int_*	0.0925	0.0616
Number of reflections with *I* > 2σ(*I*)	5911	5464
*GooF*	1.035	1.125
*R* factor on *F*^2^ > 2σ(*F*^2^)	*R*_1_ = 0.0598, *wR*_2_ = 0.1483	*R*_1_ = 0.0568, *wR*_2_ = 0.1486
*R* factor (all data)	*R*_1_ = 0.0937, *wR*_2_ = 0.1633	*R*_1_ = 0.0607, *wR*_2_ = 0.1527
Δρ_max_/Δρ_min_, *e*/Å^3^	2.639/−2.586	10.197/−9.101

**Table 2:** Selected bond distances (Å) and angles (deg.) for **I** and **II**.

	I	II
Pt-N	2.000(6), 2.005(6)	2.010(12), 2.015(12)
Pt-Cl	2.2963(18), 2.2892(18)	2.281(4), 2.301(4)
N-C_imine_	1.292(9), 1.304(9)	1.30(2), 1.300(17)
N-C_Ar_	1.443(9), 1.446(9)	1.434(17), 1.455(19)
C_imine_-C_imine_	1.425(10)	1.44(2)
Pt-N-C_imine_	114.9(5), 116.1(5)	114.0(10), 115.0(10)
Pt-N-C_Ar_	124.3(4), 127.3(5)	124.3(9), 124.6(9)
N-Pt-N	78.5(2)	79.3(5)
N-Pt-Cl	96.15(17), 94.79(17)	94.0(4), 96.3(3)
Cl-Pt-Cl	90.62(7)	90.68(15)
N-C_imine_-C_imine_-N	0.1(10)	5(2)
C_imine_-N-C_Ar_-C_orto_	80.3(9), 95.7(9)	90(2), 96.8(19)

**Table 3:** The CV data for **I**–**III** (CH_2_Cl_2_, GC, 0.1 M nBu_4_ClO_4_, C = 2·10^−3^ M, Ar, vs. Ag/AgCl/KCl (sat.)).

Compound	E^red1^_1/2_, V	I_a_/I_c_	E^red2^_p_, V
**I**	–0.40	0.92	–1.75
**II**	–0.75	0.82	–1.78
**III**	–0.71	0.92	–1.84

**Table 4:** IC50 values of **I**–**III** and CP on SKOV3 and HDF.

Cell Type	IC50 (μM)			
	I	II	III	CP
SKOV3	95	>100	12	6.5
HDF	49	>100	9	22

## Data Availability

The structure parameters of obtained compounds were deposited with the Cambridge Structural Database (CCDC Nos. 2216696 (**I**) and 2216697 (**II**); deposit@ccdc.cam.ac.uk or http://www.ccdc.cam.ac.uk/data_request/cif, accessed on 13 October 2022).

## Supplementary Materials

The following supporting information can be downloaded at: https://www.mdpi.com/article/10.3390/molecules27238565/s1, Figures S1–S5: ^1^H and ^13^C NMR spectra of **I–III**; Figures S6–S8: IR-spectra of **I**–**III**; Figures S9–S11: cyclic voltammograms of **I**, **III**, Mes-DAD(Me)_2_; Table S1: parameters of hydrogen bonds in the crystal packing in **I** and **II**.

## Sample Availability

Samples of the compounds are available from the authors.
