# Supplementary material for: α-Diimine Cisplatin Derivatives: Synthesis, Structure, Cyclic Voltammetry and Cytotoxicity
Source: Molecules. 2022 Dec 5;27(23):8565. doi: 10.3390/molecules27238565 (PMC9737600; doi:10.3390/molecules27238565)
Supplement: Supplementary file 1 [file molecules-27-08565-s001.zip › molecules-2080484-supplementary.pdf]

Supplementary Materials

α-Diimine Cisplatin Derivatives: Synthesis, Structure, Cyclic Voltammetry and Cytotoxicity

Dmitriy S. Yambulatov [1,*], Irina A. Lutsenko [1], Stanislav A. Nikolaevskii [1,*], Pavel A. Petrov [2], Ivan V. Smolyaninov [3], Irina K. Malyants [4], Victoria O. Shender [4], Mikhail A. Kiskin [1], Alexey A. Sidorov [1], Nadezhda T. Berberova [3] and Igor L. Eremenko [1]

[1] N. S. Kurnakov Institute of General and Inorganic Chemistry, Russian Academy of Sciences, 31 Leninsky Prosp., 119991 Moscow, Russia
[2] Nikolaev Institute of Inorganic Chemistry, Siberian Branch of the Russian Academy of Sciences, 630090 Novosibirsk, Russia
[3] Department of Chemistry, Astrakhan State Technical University, 16 Tatisheva Str., 414056 Astrakhan, Russia
[4] Federal Research and Clinical Center of Physical-Chemical Medicine of Federal Medical Biological Agency, 1a M. Pirogovscaya, 119435 Moscow, Russia
* Correspondence: yambulatov@yandex.ru (D.S.Y.); sanikol@igic.ras.ru (S.A.N.); Tel./Fax: +7-(495)-955-4817 (S.A.N.)

Table S1. Parameters of hydrogen bonds in the crystal packing in **I** and **II**

Hydrogen bond	Symmetry code	D – H, Å	H...A, Å	D...A, Å	D – H– A, °
			**I**		
C1-H… Cl2	x, -y+3/2, z+1/2	0.93	2.77	3.128(8)	104
C2-H… Cl1	x, -y+3/2, z+1/2	0.93	2.84	3.511(8)	130
			**II**		
C13-H13B…Cl2	x, -y+1, z-1/2	0.98	2.76	3.670(16)	154
C12-H12A…Cl1	x, -y+1, z-1/2	0.98	2.90	3.770(16)	148

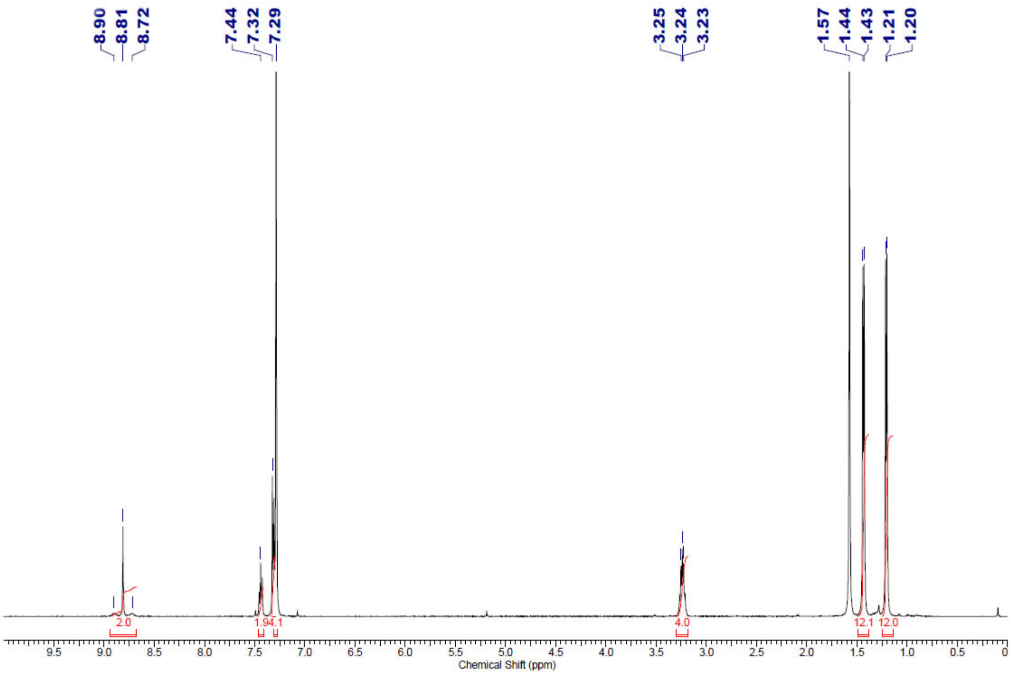

Figure S1. ¹H NMR spectrum of **I** in CDCl3.

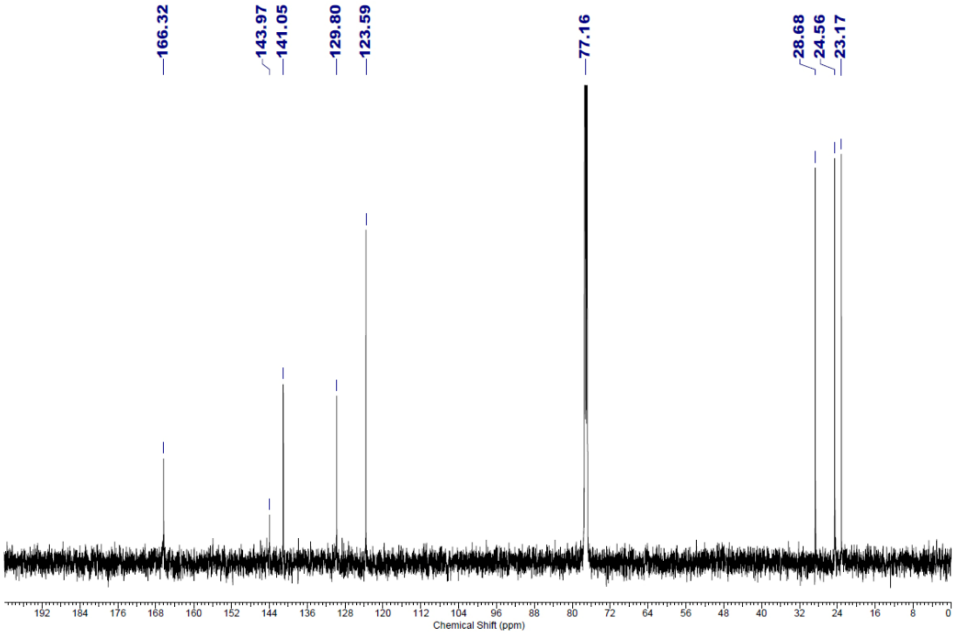

Figure **S2.** ¹³C NMR spectrum of **I** in CDCl3.

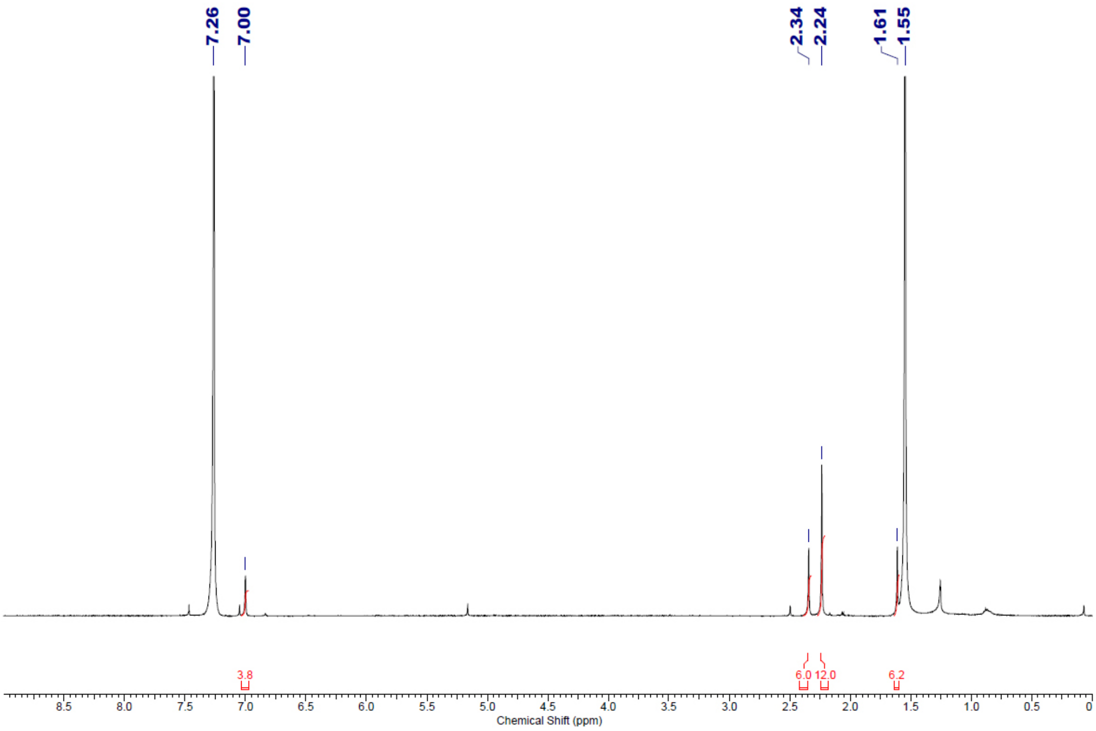

Figure S3. ^{1}H NMR spectrum of **II** in $CDCl_3$.

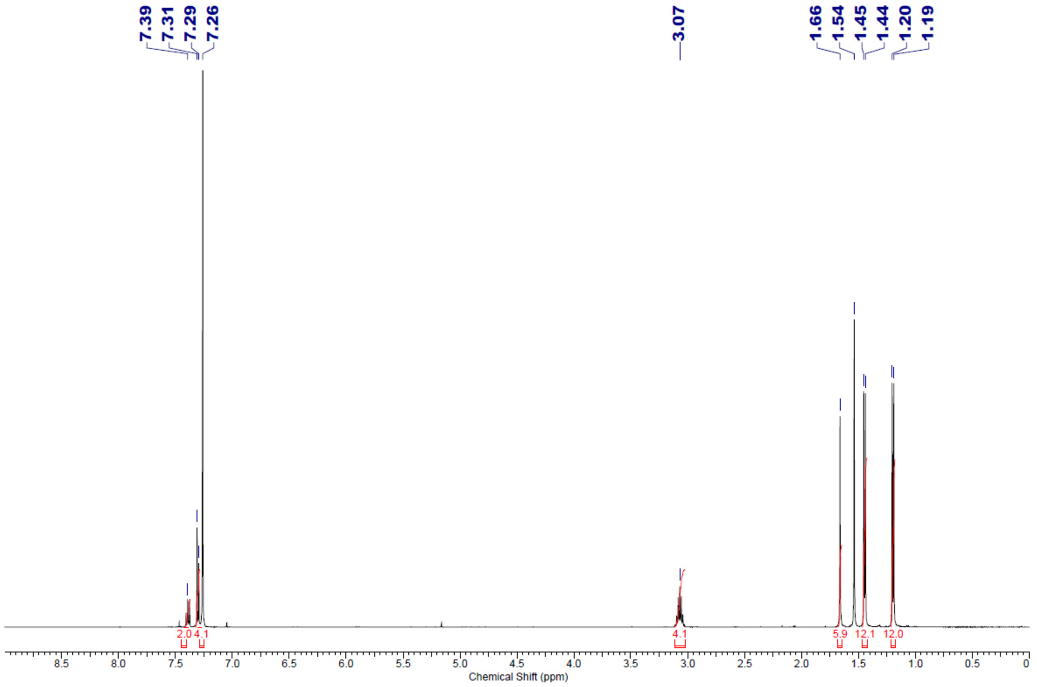

Figure S4. ^{1}H NMR spectrum of **III** in $CDCl_3$.

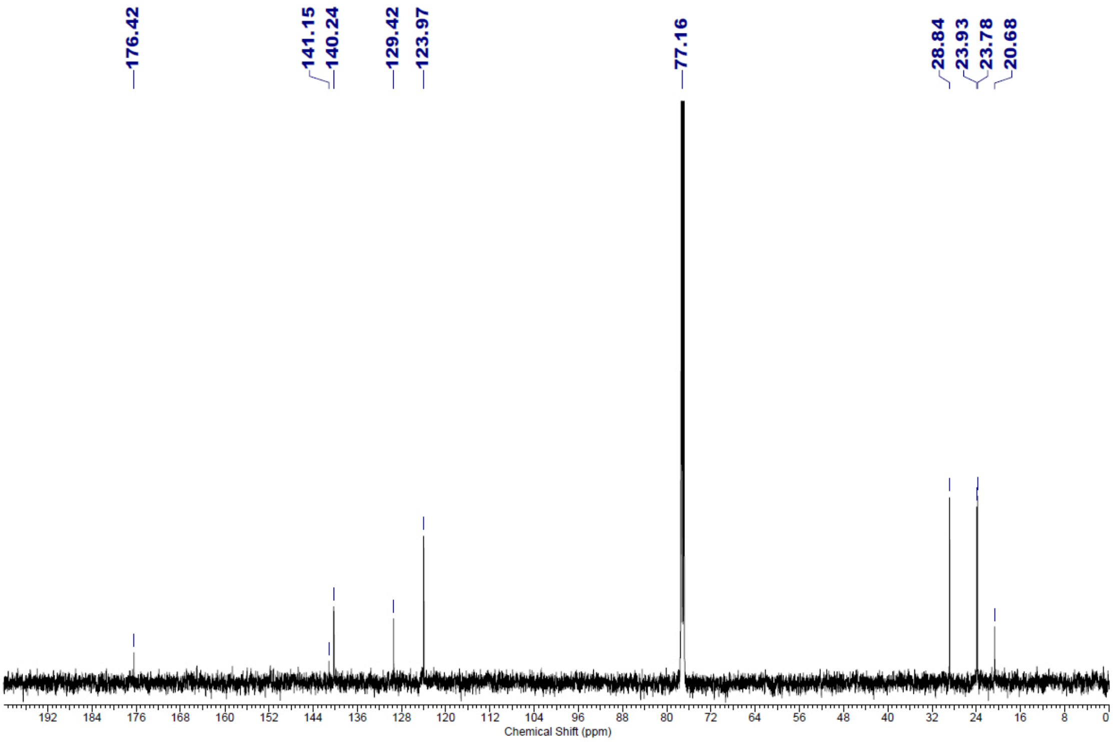

Figure S5. ^{13}C NMR spectrum of **III** in $CDCl_3$.

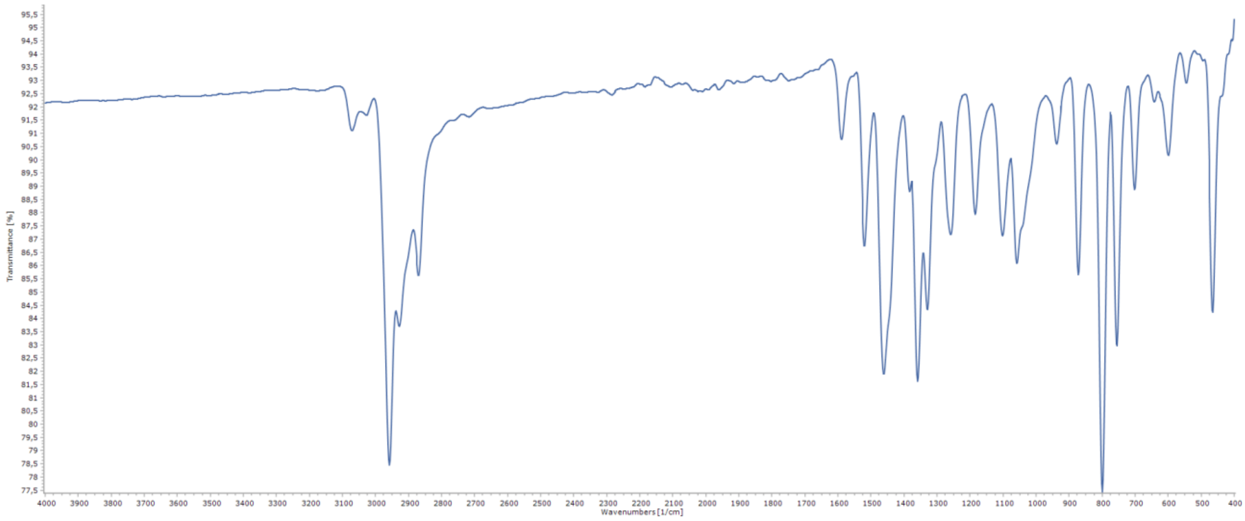

Figure S6. IR spectrum of **I**

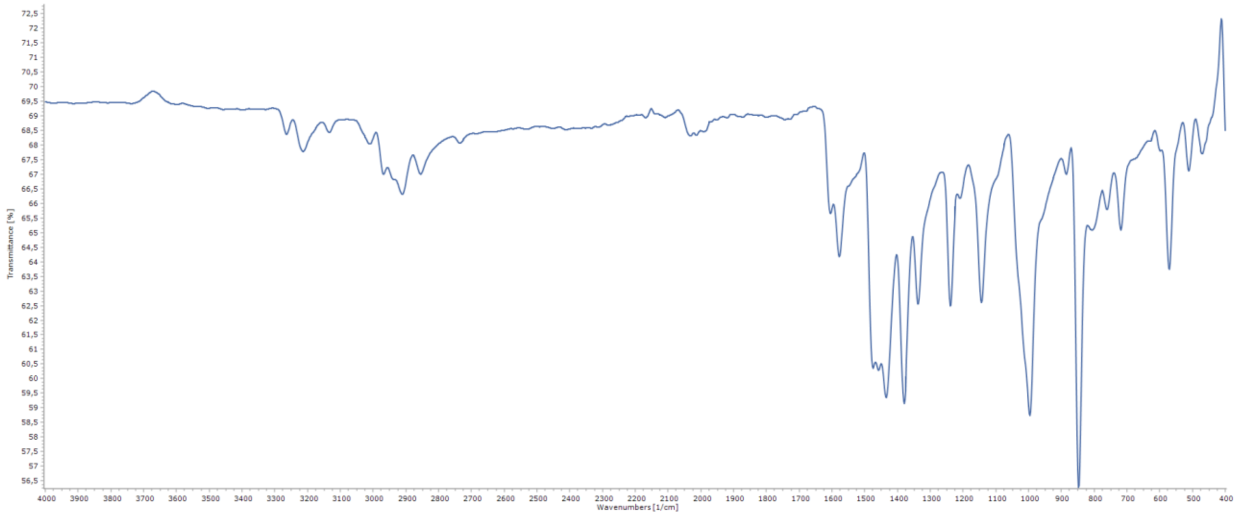

Figure S7. IR spectrum of **II**

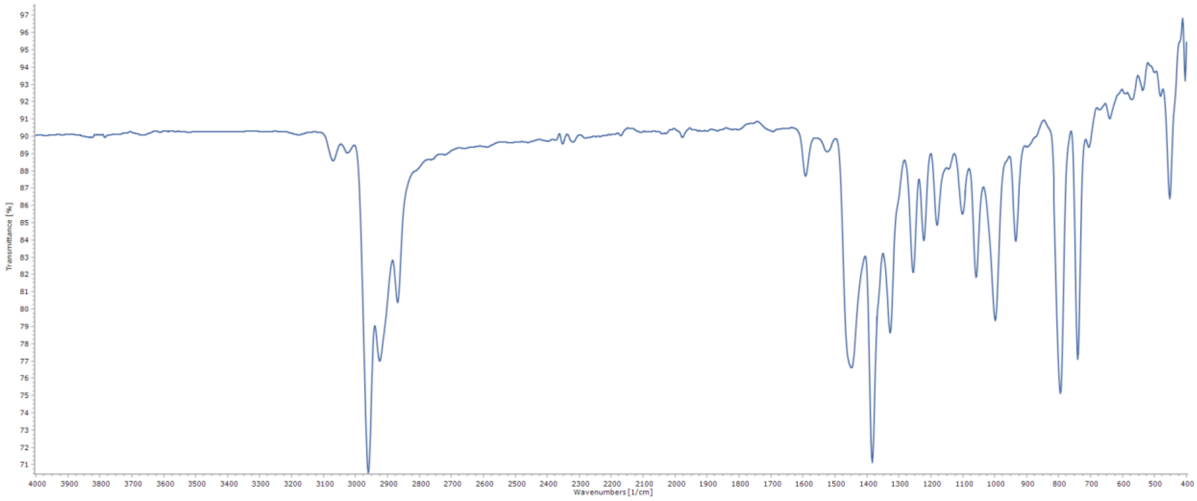

Figure S8. IR spectrum of **III**

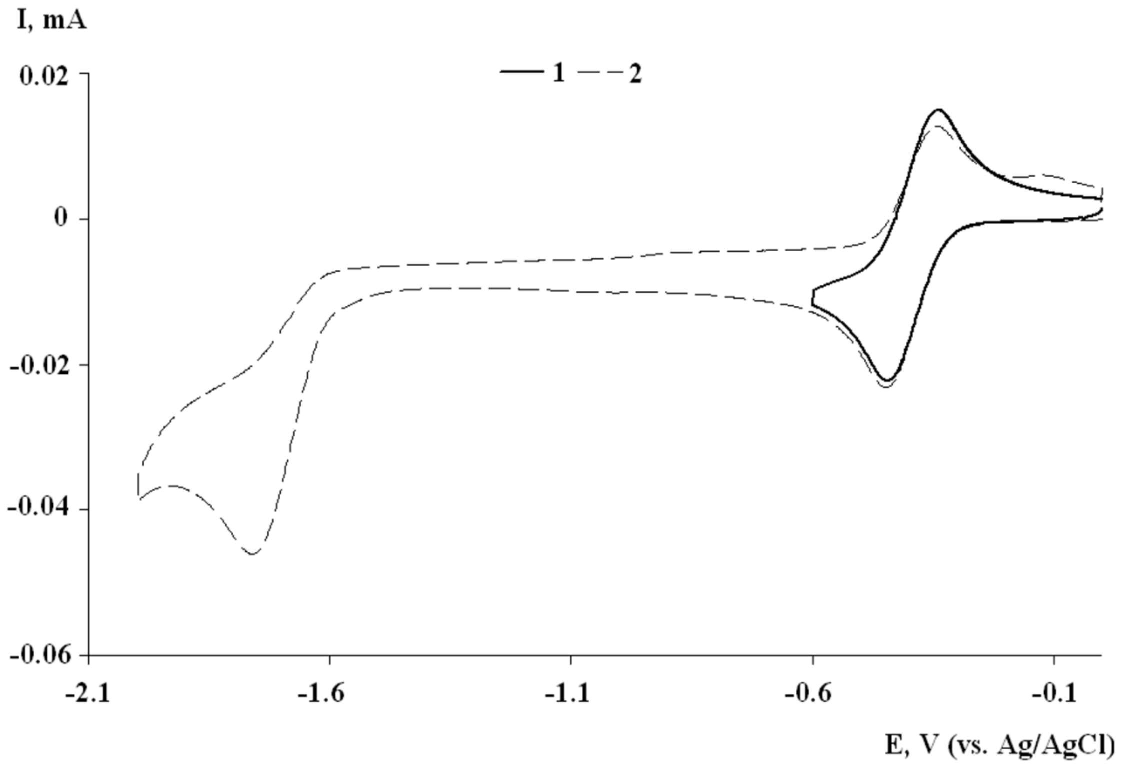

Figure S9. Cyclic voltammograms of **I**: the potential switch from 0.0 to -0.6 V (curve 1); the potential switch from 0.0 to -2.0 V (curve 2) (CH_2Cl_2, C = 2 mmol, 0.1 M TBAP, scan rate 200 mV·s^{-1}).

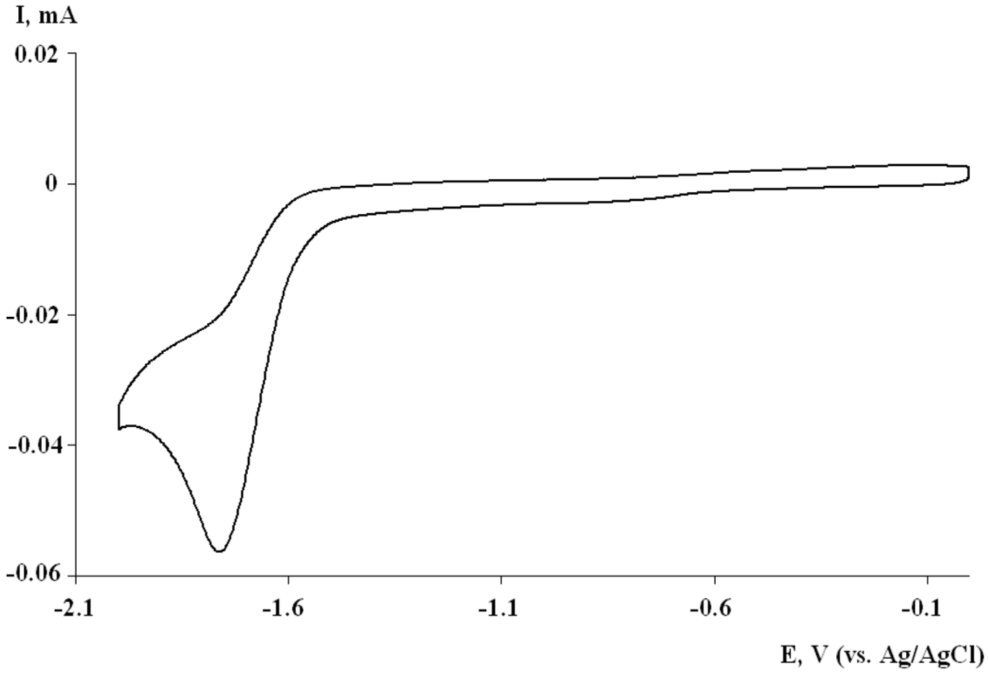

Figure S10. Cyclic voltammograms of Mes-DAD(Me)$_2$ in the potential switch from 0.0 to -2.0 V (CH_2Cl_2, C = 2 mmol, 0.1 M TBAP, scan rate 200 mV·s^{-1}).

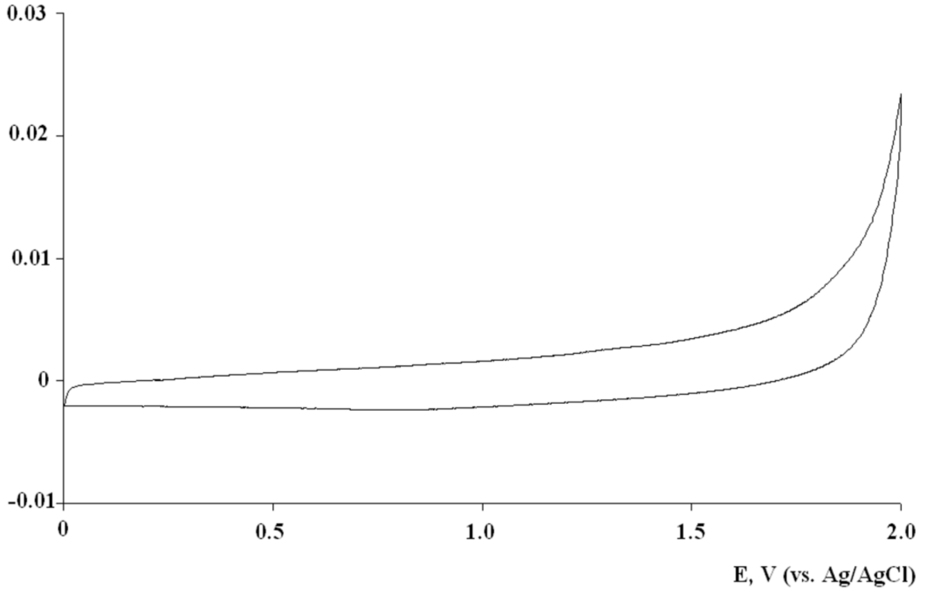

Figure S11. Cyclic voltammograms complex **III** in the potential switch from 0.0 to 2.0 V (CH_2Cl_2, C = 2 mmol, 0.1 M TBAP, scan rate 200 mV·s^{-1}).
